# Retraction Notice to: GLP2 Promotes Directed Differentiation from Osteosarcoma Cells to Osteoblasts and Inhibits Growth of Osteosarcoma Cells

**DOI:** 10.1016/j.omtn.2022.12.002

**Published:** 2022-12-11

**Authors:** Yi Lu, Dongdong Lu, Yu Hu

## Main text

(Molecular Therapy: Nucleic Acids *10*, 292–303; March 2018)

This article has been retracted at the request of the editor-in-chief. Similarities were found between images in this article and an article in *Oncotarget* (An et al., 2017, Oncotarget *25*, 49093-49109, https://doi.org/10.18632/oncotarget.17095). One author, D.L., appeared as an author of both papers. Image analysis performed by the editorial office confirmed findings of image duplication in Figure 6D of the *Molecular Therapy - Nucleic Acids* article. This reuse (and in part misrepresentation) of data without appropriate attribution represents a severe abuse of the scientific publishing system.

